# Supplementary material for: Chromothripsis during telomere crisis is independent of NHEJ, and consistent with a replicative origin
Source: Genome Res. 2019 May;29(5):737–49. doi: 10.1101/gr.240705.118 (PMC6499312; doi:10.1101/gr.240705.118)
Supplement: Supplemental Material [file supp_gr.240705.118_Supplemental_file_1.zip › contigs/annotated_contigs/DB56/contig.2.DB56_length_613_mean_cov_10.7699836868.docx]

**DB56_length_613_mean_cov_10.7699836868**

```
ATTCTATATTCCCTTTGCCTTCAGCAAGCACCTCGGCAGGTCGTGGTTTTTTTTCCTGATGGAGTGATCCAAACTTTCATTCCTGAAGG
 >chr8:101220452-101220766 - E=3e-177
GTCTGGGCCATTTGTAGTCCTGGCTGGGTTGGGCTATTGTAATTTCCCATTGACCTTAATCAGAGGGCATGGTAATACTAAGACACGCC

CTCATGGATCTCCTGCATTCCATGCATACTCTTACTTACCTCCATTGTGGAGTAGACTGATTTTGTCTTGAGAGTCCGGGTCAATCACC

CAGCCAACACTGTGACTCCATTCTTAGCCAGTTGACTTAAAGGTA|GG|TTCAAGACCAGCCTGGCCAACATGATGAAACTCCATCTCT
                                              >chr8:101211597-101211899 - E=3e-165
ACTAAAAATACAAAAAATTAGCTGGGCATGGTGGTGGGAGCCTGTAATCCCAGCTACTTGGGAGGCTGAGGCAGGAGAATTGCTTGAAC

CTGGGAGGCGGAGGTTGTAGTGAGCCAAGATCATGCCATTGCACTCCAGCCTGGGCAACAAGAGTGAAAATCCATCTCAACAACAACAA

CAAAAAAAGGCAAAGAACTTGAATAGACATTTCTCCAAAGAGGATATAGAAATAGCCCATAGGGATTGAAGAGACAGTCAA
```
